# Low-Profile Metasurface Antenna for Broadband RCS Reduction and Omnidirectional Radiation

**DOI:** 10.3390/ma19122542

**Published:** 2026-06-12

**Authors:** Liqiu Hu, Sijia Li, Kefeng Ji, Yuhao Wu, Zhiyun Zhang

**Affiliations:** Information and Navigation College, Air Force Engineering University, Xi’an 710077, China; 15797382983@163.com (L.H.); jkf0423@163.com (K.J.); kuonjiwyh@163.com (Y.W.); zzymicrowave@163.com (Z.Z.)

**Keywords:** low-profile, omnidirectional radiation, low-RCS, F-shaped feeding structure, Pancharatnam-Berry phase theory

## Abstract

A low-profile, low radar cross-section (RCS) omnidirectional metasurface antenna is investigated and proposed in this letter. The antenna consists of a top circular patch, a three-layer dielectric substrate, a full metal ground, a multi-layer polarization conversion metasurface, and four short vias for connecting the top patch to the ground. Wideband impedance matching is achieved by modifying an F-shaped feeding structure. The broadband RCS reduction is realized by loading the antenna with the polarization conversion metasurface (PCM) in an appropriate array configuration. The antenna prototype has been fabricated and measured in an anechoic chamber. Experimental results illuminated that the antenna features a low profile of 0.051λ_0_ (λ_0_ is the wavelength at 2.35 GHz) and a 10 dB impedance bandwidth of 2.11–2.62 GHz (a fractional bandwidth of 21.56%). Significantly broadband RCS reduction is achieved from 7.05 to 16.96 GHz, with a maximum reduction of –28 dB and an average reduction of –12.51 dB.

## 1. Introduction

The omnidirectional antenna can be found in many areas such as aviation communication, indoor base stations, satellite communication, navigation system, and tracking applications due to their simple structure and vast radiation pattern. Yet, the traditional omnidirectional antennas could compromise the low-scattering characteristics of the platform, significantly degrading the overall stealth. Therefore, the antenna with low radar cross-section (RCS) and omnidirectional radiation has become increasingly important for fighter aircraft.

Up to now, numerous researchers have paid attention to the low-profile omnidirectional antennas [1,2,3,4,5,6,7,8,9,10,11,12,13,14,15,16,17,18,19,20,21,22,23,24,25,26]. A low-profile, omnidirectional circularly polarized (CP) patch antenna was proposed in [1]. This design utilizes two monopole modes of a circular patch, connected to a modified ground plane via a set of conductive vias to achieve wideband impedance matching. Curved branches introduced at the edge of the circular patch excite degenerate modes (TM_01_ and TM_02_), generating circularly polarized waves. This antenna features a low profile of 0.024λ (3 mm), a 10 dB impedance bandwidth of 19.8%, and a 3 dB axial ratio bandwidth of 19.3%. It is true that the radiation performance is easily affected by the carrier due to the modified ground plane. However, the metal ground planes in these antennas are defective, which easily leads to electromagnetic leakage in practical applications and affects system compatibility. Conversely, a full metal ground plane often results in a significant increase in RCS for an omnidirectional antenna.

As a momentous scheme, the metasurfaces have been introduced to reduce the RCS of antennas [27,28,29]. Metasurfaces are ultrathin, planar artificial materials composed of subwavelength-structured unit cells, which can flexibly manipulate the amplitude, phase, polarization, and propagation direction of electromagnetic waves with unprecedented precision [30,31,32,33,34]. In [27], several resistors were loaded into the metasurface to achieve absorption. The RCS reduction can be achieved by amplitude manipulation. In [28], a transmissive polarization conversion metasurface was properly arrayed and loaded above the antenna. A multilayer transmissive metasurface was designed in [29], where the bottom layer utilizes a frequency-selective surface to achieve high transmission efficiency in the operating band and RCS reduction in two out-of-band frequency ranges. Yet, these antennas all radiate a normal beam with low RCS. Different from the existing literature, a low-profile, low-RCS omnidirectional antenna has been proposed and designed with a full-metal ground. An F-shaped feeding structure was realized to achieve Wideband impedance matching. The antenna device has been fabricated based on a 6 mm PCB board. Measurement demonstrates that the proposed antenna achieves a low profile of 0.051λ_0_ (λ_0_ is the wavelength at 2.35 GHz) and a 10 dB impedance bandwidth of 2.11–2.62 GHz (a fractional bandwidth of 21.56%). Significant RCS reduction is obtained from 7.05 to 16.96 GHz with a maximum reduction of −28 dB and an average in-band reduction of −11.3 dB. The proposed metasurface antenna has potential applications on the stealth airborne platforms.

## 2. Antenna Design

### 2.1. Antenna Configuration

**Figure 1:**
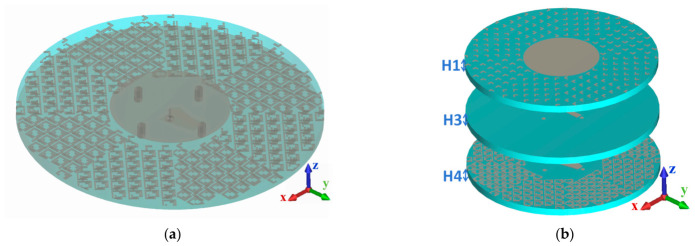

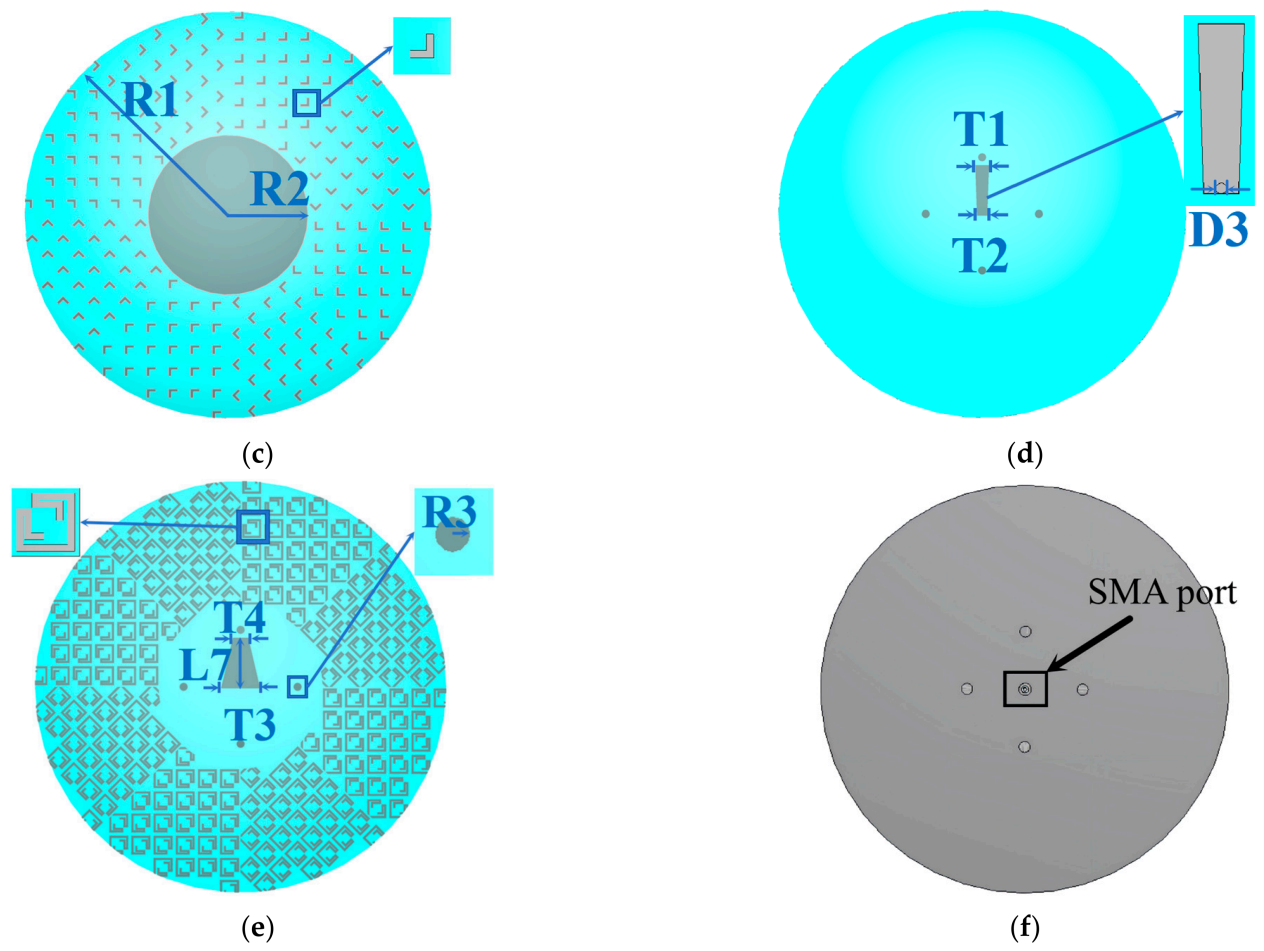
The proposed metasurface antenna. (**a**) Perspective view. (**b**) Side view. (**c**) Top layer. (**d**) Second layer. (**e**) Third layer. (**f**) Bottom layer. (R1 = 62.5, R2 = 25.6, R3 = 1.7, H1 = 1.4, H3 = 2.3, H4 = 2.3, L7 = 15.5, T1 = 4.4, T2 = 3.3, T3 = 12.4, T4 = 4.9, D3 = 1. Unit: mm).

**Figure 2:**
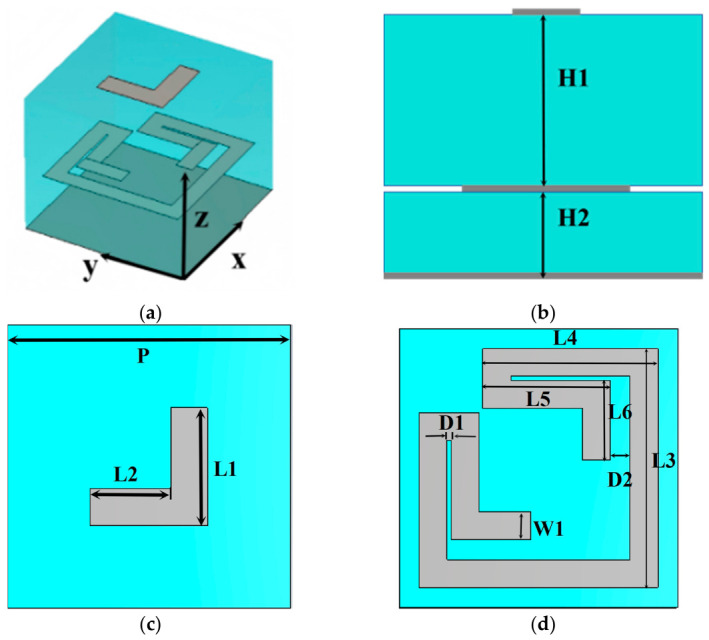
The proposed PCM. (**a**) Perspective view. (**b**) Side view. (**c**) Top layer. (**d**) Second layer. (P = 7, H2 = 3.7, L1 = 2.9, L2 = 2, L3 = 6, L4 = 4.4, L5 = 3.2, L6 = 2, D1 = 0.1, D2 = 0.5, W1 = 0.7. Unit: mm).

Figure 1 illustrates the structure of the omnidirectional antenna, which consists of three dielectric layers, a top circular patch, a full metal ground, three-layer polarization conversion metasurfaces, and four metalized vias for connecting the top patch to the ground. Figure 2 shows the configuration of the proposed polarization conversion metasurface (PCM) unit, composed of two dielectric layers and three metal patches. The proposed metasurface antenna is fabricated on a Rogers RT5880 substrate. The key material parameters are: a relative permittivity (εr) of 2.20 and a loss tangent (tanδ) of 0.001. The thickness of the PCM unit is equal to the total thickness of the antenna (H1 + H3 + H4 = H2 + H4 = 6 mm). Furthermore, the thickness of the upper dielectric layer of the PCM unit equals the combined thickness of the upper two dielectric layers of the antenna (H2 = H1 + H3 = 3.7 mm). Consequently, the upper two metallic layers of the PCM unit are, respectively, loaded onto the top and third dielectric layers of the antenna.

Figure 1c shows the top-layer structure of the antenna. In the center, there is a circular patch with radius R2. This patch is connected to the bottom ground plane through four metalized vias with a radius of R3. The top-layer metallic patches of the PCM unit are loaded around the central circular patch. As shown in Figure 1d,e, the central patches on both the second and third layers are trapezoidal metal structures, designated as Trapezoidal Patch-1 and Trapezoidal Patch-2, respectively. The length of these trapezoidal patches is L7. The other length of Trapezoidal Patch-1 is denoted as T1 and T2. Similarly, T3 and T4, respectively, denote the other length of Trapezoidal Patch-2. These two trapezoidal patches are connected by a feeding probe of an SMA port for the antenna. The second-layer metallic patches of the PCM unit are disposed around Trapezoidal Patch-2. The feeding port of the antenna is located at the bottom.

**Figure 3:**
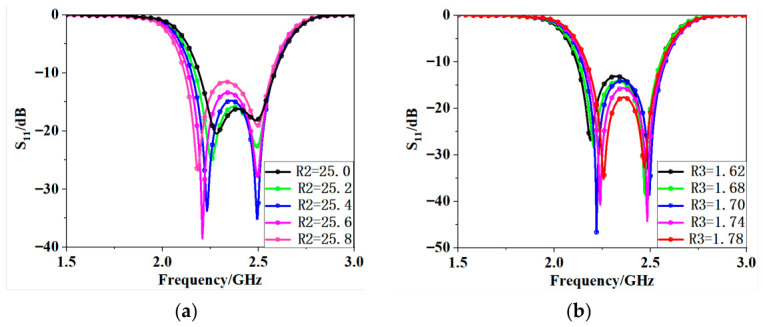
Variation in the antenna S11 parameter with (**a**) R2. (**b**) R3.

**Figure 4:**
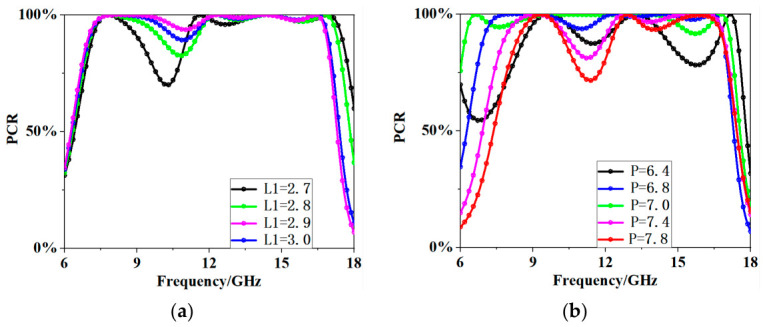
Variation in PCM PCR parameter with (**a**) L1. (**b**) P.

As shown in Figure 3, parametric sweeps of R2 and R3 were performed according to the variation in the antenna S11 parameter, and the final values were determined as R2 = 25.6 mm and R3 = 1.70 mm. Similarly, as illustrated in Figure 4, parametric sweeps of L1 and P for the PCM unit were conducted based on the variation of its polarization conversion ratio, and the optimized parameters were determined as L1 = 2.9 mm and P = 7.0 mm.

### 2.2. Feeding Structure

**Figure 5:**
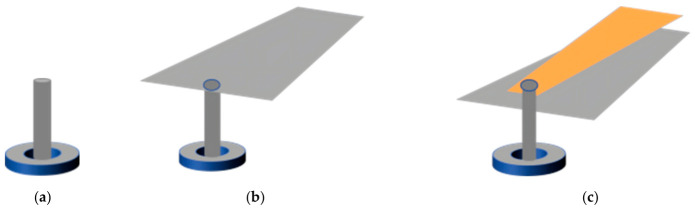
Comparison of three different feeding structures. (**a**) Structure 1. (**b**) Structure 2. (**c**) Structure 3. (Orange and gray both represent metal materials, and Structure 3 consists of two layers of metal patches).

**Figure 6:**
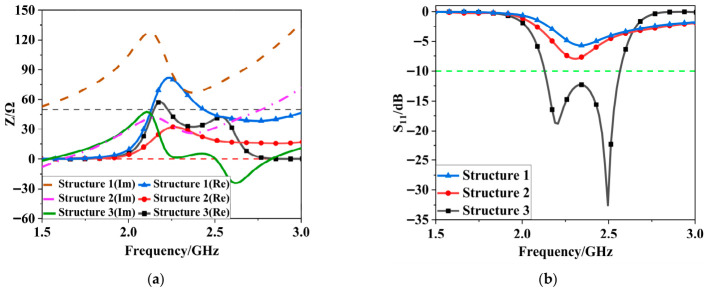
Comparison of (**a**) the equivalent impedance and (**b**) amplitude of S11 for the three feeding structures.

**Figure 7:**
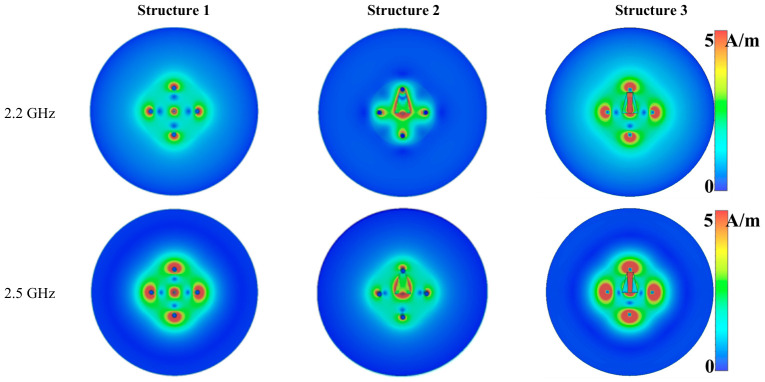
Surface current distribution of the three structures at 2.2 and 2.5 GHz.

To clarify the F-shaped feeding structure of the antenna, its design procedure and corresponding results are analyzed, as specifically illustrated in Figure 5 and Figure 6. The traditional coaxial feeding structure is shown in Figure 5a. The feeding probe directly excites the top circular patch, resulting in poor impedance matching in Figure 5a. To achieve a wide bandwidth, a multi-layer configuration of Γ- and F-shaped feeding structures was explored, as depicted in Figure 5b,c. In the design, they are denoted as Structure 2 and Structure 3. The feeding probe excites the metal patches on the intermediate layers. As shown in Figure 6a, a comparison of the real and imaginary parts of impedance reveals that the inductive reactance of the antenna gradually decreases as the number of layers increases for the three feeding structures. Observation of the surface current distribution in Figure 7 reveals that the F-shaped feed effectively enhances the surface current intensity of the antenna. Figure 6b shows a comparison of the S_11_ curves of the three structures. The results indicate that the employment of an F-shaped feed increases the resonant frequency of the antenna, converting it from a single-resonance mode to a cascade of multi-resonance modes, thereby broadening the radiation bandwidth. Therefore, F-shaped feeding of Structure 3 is employed in this paper.

### 2.3. PCM Array Design

**Figure 8:**
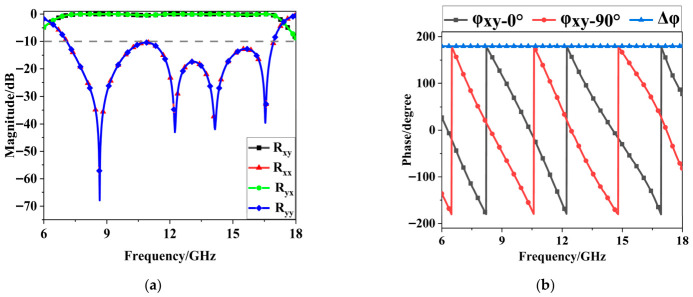
The simulated results of the reflection coefficient. (**a**) Reflection magnitude. (**b**) Reflection phase.

The simulated co-polarized and cross-polarized reflection magnitude and phase of the unit are shown in Figure 8a. The magnitude of co-polarized reflection (Rxx and Ryy) is less than −10 dB, and that of cross-polarized reflection (Ryx and Ryx) is near 0 dB from 7.05 to 16.96 GHz, which indicates that the unit achieves polarization rotation in the band.

For broadband RCS reduction based on a chessboard array, a pair of units must be constructed so that the amplitude of the cross-polarized reflection is equal and their phase difference is 180°. According to Pancharatnam–Berry phase theory [35], when a right-handed circularly polarized (RHCP) wave is incident along the -*z*-axis, the incident electric field vector ${\vec{E}}^{i}(\omega )$ and the reflected electric field vector ${\vec{E}}^{r}(\omega )$ can be expressed as:

(1) $${\vec{E}}^{i}(\omega )=E_{0}(\omega )\vec{e_{x}}-jE_{0}(\omega )\vec{e_{y}}$$

(2) $${\vec{E}}^{r}(\omega )=R_{x}E_{0}(\omega )\vec{e_{x}}+R_{y}E_{0}(\omega )\vec{e_{y}}$$

*R* denotes the reflection efficiency of the unit, and *Φ* represents the phase of the incident wave. Subsequently, the reflection coefficients *R_x_* and *R*_y_ can be expressed as follows:

(3) $$\left\{\begin{array}{c} R_{x}=\left|r_{xx}\right|e^{-j{Ф}_{xx}}+\left|r_{yx}\right|e^{-j{Ф}_{yx}}\\ R_{y}=\left|r_{yy}\right|e^{-j{Ф}_{yy}}+\left|r_{xy}\right|e^{-j{Ф}_{xy}} \end{array}\right.$$

When the unit is rotated by *φ* with respect to the *y*-axis, the incident electric field vector ${\vec{E}}^{ir}$ and the reflected electric field vector ${\vec{E}}^{rr}$ after rotation can be expressed in the *u-v* relative coordinate system as follows:

(4) $$\begin{array}{c} \left(\begin{array}{c} {\vec{E}}^{ir}(\omega )\\ {\vec{E}}^{rr}(\omega ) \end{array}\right)=E_{0}(\omega )\left(\begin{array}{cc} 1 & -j\\ R_{u} & -jR_{v} \end{array}\right)\left(\begin{array}{cc} \cos\varphi & -\sin\varphi \\ \sin\varphi & \cos\varphi \end{array}\right)\left(\begin{array}{c} \vec{e_{u}}\\ \vec{e_{v}} \end{array}\right)\\ =E_{0}(\omega )\left(\begin{array}{cc} e^{-j\varphi } & -je^{-j\varphi }\\ R_{u}e^{-j\varphi } & -jR_{v}e^{-j\varphi } \end{array}\right)\left(\begin{array}{c} \vec{e_{u}}\\ \vec{e_{v}} \end{array}\right) \end{array}$$

Since the incident wave is a circularly polarized wave, the variation trends of the reflection efficiency and reflection amplitude along the *u*-axis in the relative coordinate system are consistent with those along the *x*-axis in the original coordinate system. This indicates that the reflection amplitude *R_u_* and phase *Φ_u_* satisfy *R_u_ = R_x_* and *Φ_u_ = Φ_x_*. By analogy, the variation trends along the *v*-axis in the relative coordinate system are consistent with those along the *y*-axis in the original coordinate system. Similarly, the reflection amplitude *R_v_* and phase *Φ_v_* satisfy *R_v_ = R_y_* and *Φ_v_ = Φ_y_*. By transforming the relative coordinates back to the original coordinate system, the expression of the reflected wave ${\vec{E}}^{rr}$ after rotation in the *x*–*y* coordinate system can be obtained as follows:

(5) $$\begin{array}{c} {\vec{E}}^{rr}(\omega )=E_{0}(\omega ){\left(\begin{array}{c} R_{u}e^{-j\varphi }\\ -jR_{v}e^{-j\varphi } \end{array}\right)}^{T}\left(\begin{array}{cc} \cos\varphi & \sin\varphi \\ -\sin\varphi & \cos\varphi \end{array}\right)\left(\begin{array}{c} \vec{e_{x}}\\ \vec{e_{y}} \end{array}\right)\\ =\frac{E_{0}(w)}{2}{\left(\begin{array}{c} \left(R_{x}(w)-R_{y}(w)\right)e^{-j2\varphi }\\ R_{x}(w)+R_{y}(w) \end{array}\right)}^{T}\left(\begin{array}{c} \vec{e_{x}}+j\vec{e_{y}}\\ \vec{e_{x}}-j\vec{e_{y}} \end{array}\right) \end{array}$$

According to the above formula, it can be seen that ${\vec{E}}^{r}$ can be divided into two components, namely the left-handed circularly polarized component ${\vec{E}}_{\left(LHCP\right)}^{r}$ and the right-handed circularly polarized component ${\vec{E}}_{\left(RHCP\right)}^{r}$, as shown below:

(6) $${\vec{E}}^{r}(\omega )={{\vec{E}}^{r}}_{\left(LHCP\right)}(\omega )+{{\vec{E}}^{r}}_{\left(RHCP\right)}(\omega )$$

(7) $$\left(\begin{array}{c} {\vec{E}}_{\left(LHCP\right)}^{r}(\omega )\\ {\vec{E}}_{\left(RHCP\right)}^{r}(\omega ) \end{array}\right)=\frac{E_{0}(\omega )}{2}\left(\begin{array}{c} (\left|R_{x}(\omega )\right|e^{j{Ф}_{x}}-\left|R_{y}(\omega )\right|e^{j{Ф}_{y}})(\vec{e_{x}}+j\vec{e_{y}})e^{-j2\varphi }\\ \left(\left|R_{x}(\omega )\right|e^{j{Ф}_{x}}+\left|R_{y}(\omega )\right|e^{j{Ф}_{y}})(\vec{e_{x}}-j\vec{e_{y}}\right) \end{array}\right)$$

When the reflection efficiency and phase satisfy |*R_x_(ω)*| = |*R_y_(ω)*| = |*R(ω)*| and |Δ*Φ*| = |*Φ_x_* − *Φ_y_*| = *π*, Equation (7) can be expressed as follows:

(8) $$\begin{array}{c} \left(\begin{array}{c} {\vec{E}}_{\left(LHCP\right)}^{r}(\omega )\\ {\vec{E}}_{\left(RHCP\right)}^{r}(\omega ) \end{array}\right)=\frac{1}{2}R(\omega )E_{0}(\omega )\left(\begin{array}{c} e^{-j2\varphi }(1-e^{j(\pm \pi )})\left(\vec{e_{x}}+j\vec{e_{y}}\right)\\ (1+e^{j(\pm \pi )})\left(\vec{e_{x}}-j\vec{e_{y}}\right) \end{array}\right)\\ =R(\omega )E_{0}(\omega )\left(\begin{array}{c} \left(\vec{e_{x}}+j\vec{e_{y}}\right)e^{-j2\varphi }\\ 0 \end{array}\right) \end{array}$$

From Equations (1)–(8), it can be seen that when a right-handed circularly polarized (RHCP) wave is incident, only the left-handed circularly polarized component undergoes a phase change of −2*φ*.

Based on this principle, three array configurations are designed to reduce the radar cross-section (RCS) of antennas. As shown in Figure 6b, the phase difference is about 180° between the PCM unit cell and that with 90° rotation.

**Figure 9:**
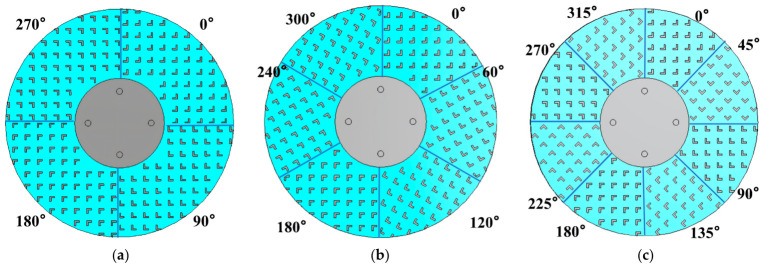
Low RCS omnidirectional metasurface antennas with three array configurations. (**a**) Configuration 1. (**b**) Configuration 2. (**c**) Configuration 3.

**Figure 10:**
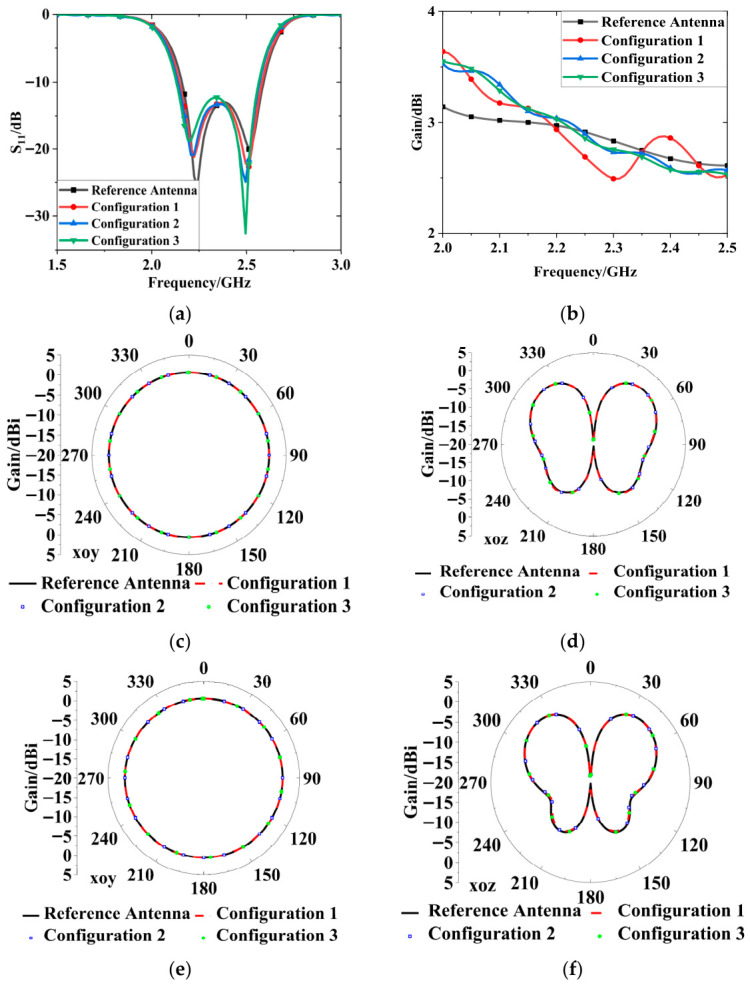
Comparison of radiation performance for the metasurface antenna with three configurations of PCM array and the reference antenna. (**a**) Amplitude of S11. (**b**) Gain. Radiation patterns in (**c**) xoy-plane at 2.2 GHz, (**d**) xoz-plane at 2.2 GHz, (**e**) xoy-plane at 2.5 GHz, and (**f**) xoz-plane at 2.5 GHz.

**Figure 11:**
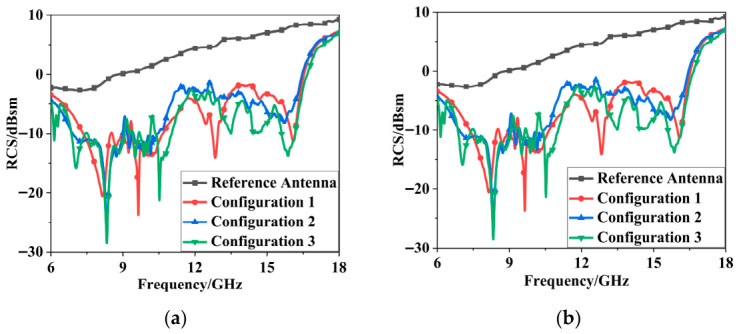
Simulated monostatic RCS of metasurface antennas and reference antenna for normal incidence with (**a**) x-polarization and (**b**) y-polarization.

**Figure 12:**
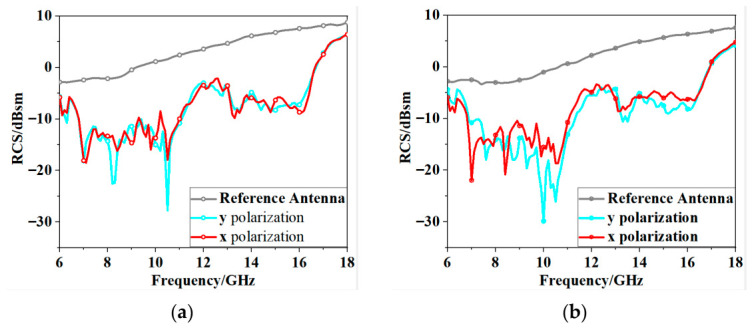
RCS simulation results under oblique incidence: (**a**) 15° and (**b**) 30°.

**Figure 13:**
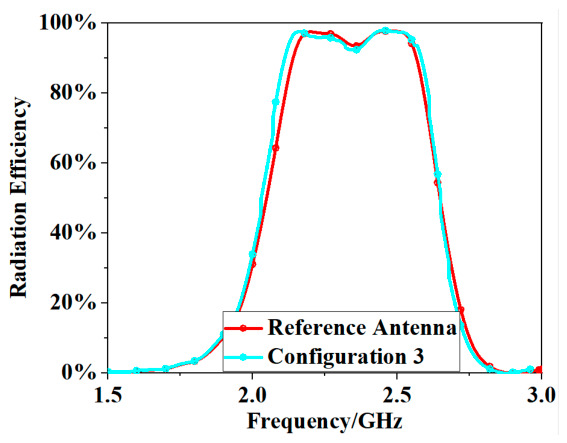
Radiation efficiency of the reference antenna and configuration 3.

**Figure 14:**
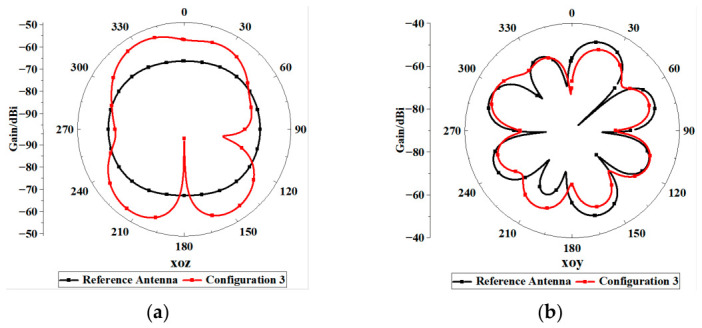
Cross-polarization level of the reference antenna and configuration 3 (**a**) xoz-plane and (**b**) xoy-plane at 2.2 GHz.

As shown in Figure 9, three configurations of omnidirectional antennas loaded with the PCM array are designed in this work, namely the chessboard array, the hexagonal array, and the octagonal array. Figure 10 illustrates that the characteristics of impedance matching, gain, and radiation patterns are hardly affected by PCM with the three array configurations. The monostatic RCS results of the antennas are presented in Figure 11. The calculated values of average RCS reduction between the reference and the metasurface antennas are listed in Table 1 in the band from 7.05 to 16.96 GHz. The metasurface antennas achieved average RCS reductions of 11.45, 10.57, and 12.52 dB under x-polarized incidence and 11.30, 10.48, and 12.28 dB under y-polarized incidence. It can be found that the most RCS reduction can be obtained for the metasurface antenna with configuration 3. In addition, simulations were performed to investigate the RCS reduction performance of the proposed antenna at oblique incidences of 15° and 30°. As illustrated in Figure 12, favorable RCS reduction characteristics are still maintained under oblique incidence conditions. To verify that PCM has little effect on the radiation performance of the antenna, we compared the radiation efficiency of the reference antenna and configuration 3. As shown in Figure 13, the radiation efficiency of the omnidirectional antenna does not change significantly after loading PCM. As illustrated in Figure 14, the cross-polarized radiation pattern of the antenna at 2.2 GHz is adopted for validation. It is shown that PCM has no obvious effect on increasing the cross-polarization level of the antenna.

## 3. Measured Results

**Figure 15:**
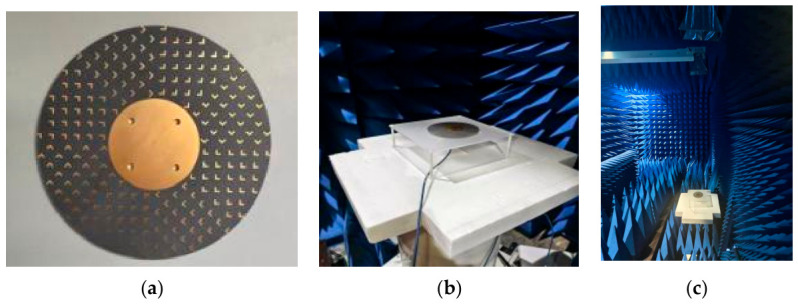
Prototype of the proposed metasurface antenna loaded with PCM array of configuration 3. (**a**) Top view. (**b**) Radiation measurement in an anechoic chamber. (**c**) Scattering measurement in an anechoic chamber.

**Figure 16:**
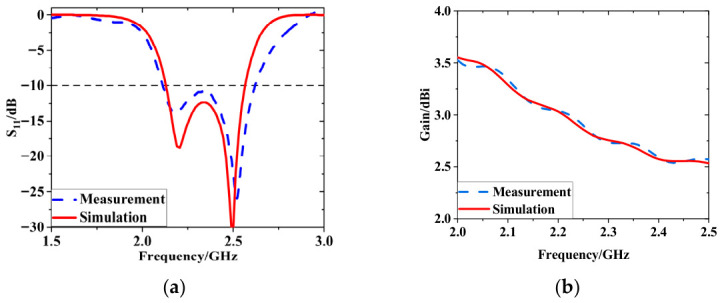
Measured and simulated results of (**a**) amplitude of S11 and (**b**) gain.

**Figure 17:**
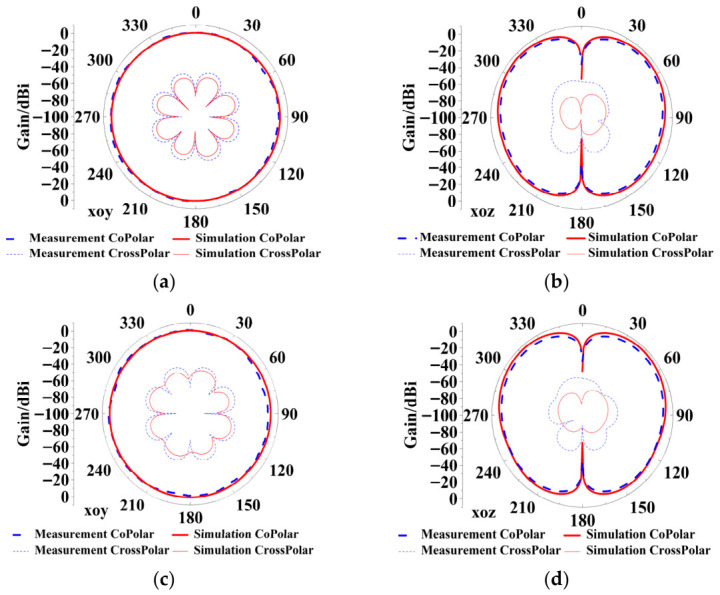
Measured and simulated radiation patterns of the proposed metasurface antenna loaded with a PCM array of configuration 3. Radiation patterns in (**a**) xoy-plane and (**b**) xoz-plane at 2.2 GHz. Radiation patterns in (**c**) xoy-plane and (**d**) xoz-plane at 2.5 GHz.

**Figure 18:**
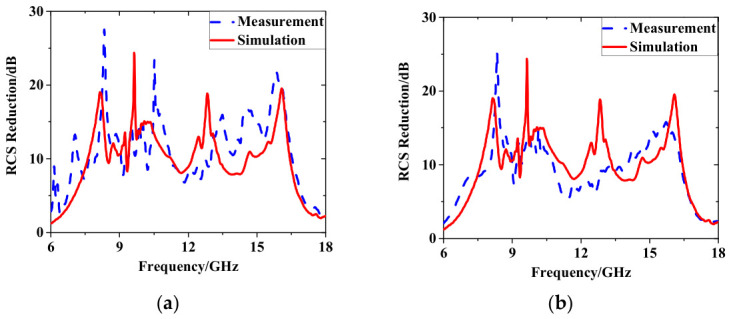
Measured and simulated RCS Reduction. (**a**) x-polarization. (**b**) y-polarization.

As shown in Figure 15, a prototype of the proposed metasurface antenna loaded with a PCM array of configuration 3 has been fabricated using PCB technology. The metasurface antenna with dimensions of π × 62.5^2^ mm^2^ × 6 mm was measured in an anechoic chamber. Figure 16a,b presents the measured and simulated results of S-parameters and gain of the antenna. The experimental results indicate that a working bandwidth covering 2.11–2.62 GHz (a fractional bandwidth of 21.56%) can be obtained with an amplitude of S11 less than −10 dB, and the gain is more than 2.5 dB for the proposed metasurface antenna. The measurement is in proper agreement with the simulation. The measured radiation patterns at the two resonance frequencies of 2.2 and 2.5 GHz are given in Figure 17. The measured results indicate that the antenna exhibits low cross-polarized components of less than −40 dB and the favorable omnidirectional characteristics with a gain variation of less than 3 dB. From the measured results of RCS reduction in Figure 18, we can obviously see that the RCS reduction above 8 dB can be achieved from 7.05 to 16.96 GHz for the metasurface antenna in normal incidences with x- and y-polarization.

A comparison is summarized in Table 2 between the proposed metasurface antenna and several existing low-profile, low RCS antennas [36,37,38,39,40,41,42,43,44,45]. Compared with antennas in [37,39,40,41,42,44], the proposed design achieves an ultra-low profile of 0.05λ_0_. The full metal ground of the proposed antenna decreases the effect of the carrier compared with the antenna in [41]. As shown in Table 2, this metasurface antenna successfully realizes the omnidirectional radiation and RCS with the advantage of ultra-low profile, a relatively small gain variation, and low cross-polarized components.

## 4. Conclusions

In this letter, we present a low-profile, broadband, low RCS omnidirectional metasurface antenna. By feeding the metal patch on the intermediate layer of the antenna, a low-profile height of 6 mm is successfully achieved. The broadband RCS reduction is realized by loading the antenna with a polarization conversion metasurface array in an octagonal configuration. Measurement and simulation demonstrated that the proposed metasurface antenna exhibits advantages of omnidirectional radiation, low RCS, stable gain, and low cross-polarized components. Owing to its characteristics, this metasurface antenna offers a potential application on a stealth aircraft.

## Figures and Tables

**Table 1:** Average RCS reduction.

Configurations	Average RCS Reduction from 7.05 to 16.96 GHz	
	x-Polarization	y-Polarization
Configuration 1	11.45	11.30
Configuration 2	10.57	10.48
Configuration 3	12.52	12.28

**Table 2:** Comparison between the proposed metasurface antenna.

Ref.	Size (λ_0_^3^)	Bandwidth (GHz)	Full Ground	RCS Reduction (GHz)	Omnidirectional Radiation	Polarization for RCS Reduction	Azimuthal Gain (dBi)
[36]	0.42 × 0.42 × 0.03	2.05–2.75 (29.3%)	√	×	√	×	1.5
[37]	π × 0.32^2^ × 0.06	3.2–4.0 (22.2%)	√	3.0–7.0	√	Dual-polarization	4.5
[38]	π × 0.35^2^ × 0.009	1.95–2.62 (29.3%)	√	×	√	×	3.2
[39]	0.85 × 0.85 × 0.1	VHF-UHF (1700%)	√	7.2–17.2	√	Dual-polarization	2.0
[40]	π × 0.55^2^ × 0.04	Dual-band (18.5%/23.6%)	√	×	√	×	4.6/6.8
[41]	π × 0.36^2^ × 0.05	6.38–17.67 (85.6%)	√	×	×	×	×
[42]	π × 0.15^2^ × 0.04	1.66–5.04 (7.2%)	√	×	√	×	1.55
[43]	0.9 × 0.9 × 0.07	1.66–5.04 (95.8%)	√	×	√	×	5.8
[44]	π × 0.35^2^ × 0.01	2.99–3.01 (1%)	√	2.99–3.01	√	Single-polarization	5.7
[45]	π × 1.22^2^ × 0.45	1.6–3.8 (81.5%)	×	×	×	×	6.1
Our design	π × 0.51^2^ × 0.05	2.11–2.6 (21.56%)	√	7.05–16.96	√	Dual-polarization	3.5

λ_0_ is the wavelength at the center frequency.

## Data Availability

The original contributions presented in this study are included in the article. Further inquiries can be directed to the corresponding author.

## References

[1] Pan Y.M., Zheng S.Y., Hu B.J. (2014). Wideband and Low-Profile Omnidirectional Circularly Polarized Patch Antenna. IEEE Trans. Antennas Propag..

[2] Tai J.-H., Chi Y.-J., Chen F.-C. (2026). A Low-Profile Omnidirectional Circularly Polarized Antenna Using Integrated Patch and CRLH Loop Structures. Sci. Rep..

[3] Wu G.B., Dai J.Y., Shum K.M., Chan K.F., Cheng Q., Cui T.J., Chan C.H. (2023). A universal metasurface antenna to manipulate all fundamental characteristics of electromagnetic waves. Nat. Commun..

[4] Sun L., Sun B.-H., Zhang G.-X. (2017). Low-Profile Omnidirectional Antenna with Dual Polarizations for 2.4 GHz WLAN Applications. Prog. Electromagn. Res. Lett..

[5] Yu L., Song J., Gao Y., He K., Gao F. (2017). Low-Profile Dual-Polarized Omnidirectional Antenna for Broadband Indoor Distributed Antenna System. Prog. Electromagn. Res. Lett..

[6] Smith D.R., Padilla W.J., Vier D.C., Nemat-Nasser S.C., Schultz S. (2000). Composite medium with simultaneously negative permeability and permittivity. Phys. Rev. Lett..

[7] Alù A., Engheta N. (2009). Cloaking a sensor. Phys. Rev. Lett..

[8] Cui T.J., Qi M.Q., Wan X., Zhao J., Cheng Q. (2014). Coding metamaterials, digital metamaterials and programmable metamaterials. Light Sci. Appl..

[9] Rana S., Jain P. (2023). Design of Low-Profile High-Gain Wideband Circularly Polarized Low RCS Single-Layer Metasurface Antenna Using Characteristics Mode Analysis. Int. J. Microw. Wirel. Technol..

[10] Cui Y., Qi C., Li R. (2019). A Low-Profile Broadband Quad-Polarization Reconfigurable Omnidirectional Antenna. IEEE Trans. Antennas Propag..

[11] Wen S., Xu Y., Dong Y. (2021). Low-Profile Wideband Omnidirectional Antenna for 4G/5G Indoor Base Station Application Based on Multiple Resonances. IEEE Antennas Wirel. Propag. Lett..

[12] Chen J., Yang F., Zhao S.-J., Shi X.-B., Huang M., Yang S.-W. (2024). Airborne Ultra-Low Profile Ultra-Wideband Omnidirectional Antenna Based on Tightly Coupled Arrays. J. Electron. Sci. Technol..

[13] Li C., Zheng Z. (2025). A Low-Profile and Compact Omnidirectional Antenna for Indoor Base Station Using Property-Matched Dielectric Materials. Microw. Opt. Technol. Lett..

[14] Nguyen-Trong N., Piotrowski A., Kaufmann T., Fumeaux C. (2016). Low-Profile Wideband Monopolar UHF Antennas for Integration onto Vehicles and Helmets. IEEE Trans. Antennas Propag..

[15] Yang L., Zhang Z.-Y., Fu G., Zhang Y.-X., Li Y. (2014). A Novel Low-Profile Quadripod Kettle Antenna with Enhanced Bandwidth. Prog. Electromagn. Res..

[16] Jiang H., Xue Z., Leng M., Li W., Ren W. (2018). Wideband Partially Reflecting Surface Antenna with Broadband RCS Reduction. IET Microw. Antennas Propag..

[17] Zürcher J.-F. (2013). TOLPA: Tripod Omnidirectional Low Profile Antenna—A Vertically Polarized Antenna with 90% Bandwidth. Microw. Opt. Technol. Lett..

[18] Guo Y., Zhao J., Hui W., Xie K., Hou Q., Zhao X. (2023). Low-Profile Omnidirectional Antenna with Broad Bandwidth and High Gain. Microw. Opt. Technol. Lett..

[19] Xu C.-C., Duan Z. (2023). A Wideband and Low-Profile Omnidirectional Circularly Polarized Antenna. Microw. Opt. Technol. Lett..

[20] Wu Y., Sun H. (2024). A Low-Profile Wideband Omnidirectional Antenna with Reconfigurable Tri-Polarization Diversity. AEU Int. J. Electron. Commun..

[21] Wu J., Sarabandi K. (2017). Compact Omnidirectional Circularly Polarized Antenna. IEEE Trans. Antennas Propag..

[22] Cai Y.-M., Gao S., Yin Y.-Z., Li W.-T., Luo Q. (2016). Compact-Size Low-Profile Wideband Circularly Polarized Omnidirectional Patch Antenna with Reconfigurable Polarizations. IEEE Trans. Antennas Propag..

[23] Peng J.-D., Fu D.-Q., Ye L.-H., Shi X. (2022). Ultra-Wideband Vertically Polarized Omnidirectional Antenna with Dual Metal Loops. Front. Phys..

[24] Modi A.Y., Balanis C.A., Birtcher C.R., Shaman H.N. (2019). New Class of RCS-Reduction Metasurfaces Based on Scattering Cancellation Using Array Theory. IEEE Trans. Antennas Propag..

[25] Jia Y., Liu Y., Guo Y.J., Li K., Gong S. (2017). A Dual-Patch Polarization Rotation Reflective Surface and Its Application to Ultra-Wideband RCS Reduction. IEEE Trans. Antennas Propag..

[26] Knott E.F., Shaeffer J.F., Tuley M.T. (2004). Radar Cross Section.

[27] Pan W., Huang C., Chen P., Ma X., Hu C., Luo X. (2014). A Low-RCS and High-Gain Partially Reflecting Surface Antenna. IEEE Trans. Antennas Propag..

[28] Pang Y., Li Y., Qu B., Yan M., Wang J., Qu S., Xu Z. (2020). Wideband RCS Reduction Metasurface with a Transmission Window. IEEE Trans. Antennas Propag..

[29] Yan S., Zhai X., Ren H., Zhang J. (2024). A Low-Profile Dual-Polarized Omnidirectional Antenna for WLAN/UWB Applications. IEEE Antennas Wirel. Propag. Lett..

[30] Liu X., Li S., He C., Li Z., Huang G., Cao X. (2023). Multiple Orbital Angular Momentum Beams with High-Purity of Transmission-Coding Metasurface. Adv. Theory Simul..

[31] Li S., Li Z., Huang G., Liu X., Li R., Cao X. (2022). Digital Coding Transmissive Metasurface for Multi-OAM-Beam. Front. Phys..

[32] Li S., Han B., Li Z., Liu X., Huang G., Li R., Cao X. (2022). Transmissive Coding Metasurface with Dual-Circularly Polarized Multi-Beam. Opt. Express.

[33] Li S., Li Z., Han B., Huang G., Liu X., Yang H., Cao X. (2022). Multifunctional Coding Metasurface with Left and Right Circularly Polarized and Multiple Beams. Front. Mater..

[34] Li S., Li Z., Liu X., He C., Huang G., Li R., Cao X. (2023). Transmissive Digital Coding Metasurfaces for Polarization-Dependent Dual-Mode Quad Orbital Angular Momentum Beams. ACS Appl. Mater. Interfaces.

[35] Zhang L., Liu S., Li L., Cui T. (2017). Spin-controlled multiple pencil beams and vortex beams with different polarizations generated by Pancharatnam–Berry coding metasurfaces. ACS Appl. Mater. Interfaces.

[36] Mei P., Lin X.Q., Yu J.W., Zhang P.C., Boukarkar A. (2018). A Low Radar Cross Section and Low Profile Antenna Co-Designed with Absorbent Frequency Selective Radome. IEEE Trans. Antennas Propag..

[37] Ren J., Gong S., Jiang W. (2018). Low-RCS Monopolar Patch Antenna Based on Dual-ring Metamaterial Absorber. IEEE Antennas Wirel. Propag. Lett..

[38] Wu S., Shang F. (2023). 5G Indoor Base Station Application: Low Profile Broadband Horizontally Polarized Omnidirectional Antenna. Prog. Electromagn. Res. C.

[39] Wang C., Yuan B., Shi W., Mao J. (2020). Low-Profile Broadband Plasma Antenna for Naval Communications in VHF and UHF Bands. IEEE Trans. Antennas Propag..

[40] Ke Y.H., Zhou J., Chen J.X. (2025). Dual-Band Dual-Polarized Omnidirectional MIMO Antenna with Ultra-Low Profile for Vehicular Communications. IEEE Trans. Veh. Technol..

[41] Hua C., Liu J., Yu Y., Ren W., Shen Z. (2024). Low-Profile and Wideband Surface-Wave Antenna of Conical Beam for UAV Applications. IEEE Antennas Wirel. Propag. Lett..

[42] Shi T., Wen Y., Wang H., Tang M.C., Yuan X. (2024). Low-Profile, Pattern-Reconfigurable, Electrically Small Antenna Based on Equivalent Even and Odd Modes. IEEE Antennas Wirel. Propag. Lett..

[43] Zhang J., Huang J., Jiang H., Ye L.H., Li J.F., Zhang X. (2024). Ultrawideband Vertically Polarized Omnidirectional Periodic Patch-Inspired Antenna with Low Profile. IEEE Trans. Antennas Propag..

[44] Luo J., Geng P., Li B., Jia Y., Liu Y. Low Scattering Omnidirectional Microstrip Antenna Design Based on Characteristic Mode Theory. Proceedings of the 2025 IEEE Applied Computational Electromagnetics Society Symposium (ACES).

[45] Lamkaddem A., Merino E., El Yousfi A., Gonzalez-Posadas V., Segovia-Vargas D. Broadband Dual-Polarized Vivaldi Base Station Array Antenna for 5G Smart Networks. Proceedings of the 2023 International Workshop on Antenna Technology (iWAT).

